# Komplexes Beckentrauma: Eine Falldarstellung unter besonderer Berücksichtigung der urogenitalen Begleitverletzung

**DOI:** 10.1007/s00113-022-01170-3

**Published:** 2022-04-05

**Authors:** David Koppe, Jana Pretzer, Peter Heumann, Katharina Salmoukas, Carlo Dietl, Moritz Goll, Axel Ekkernkamp

**Affiliations:** 1 Klinik für Unfallchirurgie und Orthopädie, Schwerpunkt Wirbelsäulen- und Beckenchirurgie, BG Klinikum Unfallkrankenhaus Berlin gGmbH, Warener Str. 7, 12683 Berlin, Deutschland; 2 Klinik für Urologie und Neuro-Urologie, BG Klinikum Unfallkrankenhaus Berlin gGmbH, Berlin, Deutschland

**Keywords:** Beckenringfraktur, Beckentrauma, Urogenitalsystem, Blasenverletzung, Nervenverletzung, Pelvic ring fracture, Pelvic trauma, Genitourinary system, Bladder injury, Nerve injury

## Abstract

Die folgende Kasuistik zeigt einen jungen Mann, der sich im Rahmen eines Verkehrsunfalles ein komplexes Beckentrauma mit schwerer Begleitverletzung der ableitenden Harnwege sowie ausgeprägter Begleitschädigung des Plexus lumbosacralis zuzog. Zur operativen Versorgung der Becken- und Blasenverletzung waren mehrere aufwendige Eingriffe nötig. Auf Grundlage eines Infektes kam es schließlich zur Wundheilungsstörung mit der Notwendigkeit einer Lappenplastik. Der Fall verdeutlicht die Komplexität dieser Verletzung und belegt die Notwendigkeit der interdisziplinären individualisierten Behandlung.

## Abstract (en)

The following case report shows a young male patient with a complex pelvic trauma due to a traffic accident. In addition to the pelvic ring fracture, he also suffered a severe accompanying injury to the efferent urinary tract in combination with extensive damage of the lumbosacral plexus. Multiple extensive operations were necessary to address the C3 fracture of the pelvic ring and the bladder injury. Due to an infection a wound healing disorder subsequently occurred, which necessitated a myocutaneous flap plasty. This case highlights the complexity of this injury and confirms the necessity for an interdisciplinary individualized treatment.

## Einleitung

Das komplexe Beckentrauma ist eine seltene Verletzung, die selbst in Schwerpunktkliniken nicht häufig anzutreffen ist. Die Begrifflichkeit des komplexen Beckentraumas ist definiert als Verletzung des knöchernen Beckenringes in Verbindung mit Begleitverletzungen der großen Gefäße, Nerven und oder inneren Organe der peripelvinen Region. Zusätzlich werden die offenen Beckenfrakturen als auch solche mit schwerem geschlossenen Weichteilschaden (z. B. Décollement-Verletzungen) dazugezählt [3]. Die eigene Begrifflichkeit ist zurückzuführen auf die geringe Inzidenz dieser schweren Verletzung (7 % aller Beckenfrakturen) und die hohe Mortalität, die damit einhergeht (18 %) [5]. Verletzungen der Gefäße finden sich hier in bis zu 27 % der Fälle. Nervenschäden sind in bis zu 50 %, Verletzungen der Blase in 25 % und die der Urethra in 15 % der Fälle anzutreffen [2, 6, 8, 12, 13].

## Anamnese

Die folgende Falldarstellung beschreibt einen 25-jährigen Patienten, der als Fahrradfahrer von einer Straßenbahn erfasst wurde. Bei Eintreffen des Notarztes war er unter der Straßenbahn eingeklemmt. Nach technischer Rettung wurde der Patient analgosediert, intubiert und beatmet. Der mittlerweile kreislaufinstabile Patient wurde bei klinisch instabilem Becken mit einem Beckengurt versorgt und in unsere Klinik verbracht.

## Klinischer Befund

Der Patient war bei Ankunft bereits analgosediert, intubiert und beatmet. Bei der klinischen Untersuchung zeigte sich eine ausgedehnte Prellmarke der rechten Flanke. Im hausinternen Vorgehen wird im Falle eines korrekt anliegenden Beckengurtes dieser im Rahmen des Primary Survey nicht erneut geöffnet und das Becken klinisch nicht auf Stabilität untersucht. Im Ultraschall (FAST) ließ sich keine freie Flüssigkeit nachweisen. Zum neurologischen Status am Unfallort gab es keine Informationen.

## Diagnose

**Figure 1:**
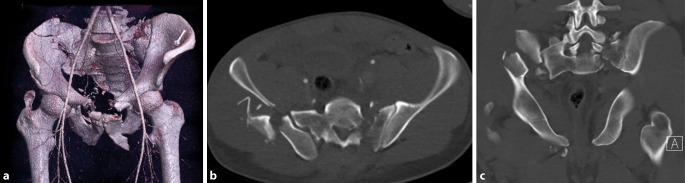

Im Polytrauma-CT bestätigte sich eine komplexe Beckenringverletzung Typ C3 nach Tile mit zum einen transpubischer Instabilität beidseits und transforaminaler Sakrumfraktur links und zum anderen mehrfragmentärer Beckenschaufelfraktur der rechten Seite mit Loslösung des gesamten Acetabulums aus dem knöchernen Verbund. Klinisch imponierte außerdem Blut am Meatus. Bereits im Polytrauma-CT wurde der Verdacht auf einen Kontrastmittelaustritt aus der blasenhalsnahen Urethra gestellt (Abb. 1; [3]).

Durch die urologischen Kollegen wurde ein transurethraler Katheter unter retrograder Darstellung der Urethra eingelegt. Nach regelrechter Kathetereinlage mit retrograder Auffüllung der Harnblase zeigte sich eine ausgeprägte Harnblasentamponade; ein Harnblasenextravasat konnte bei diffuser Blutung und fleckigem KM-Nachweis in Projektion auf das kleine Becken nicht ausgeschlossen werden. Nach Erreichen einer akzeptablen Blasenkapazität wurde ein suprapubischer Fistelkatheter angelegt. Anschließend entleerte sich zunächst klarer Urin mit akzeptablen Stundenportionen (80–100 ml/h). Ein „re-alignment“ der Harnröhre (Wiederherstellung der Harnröhrenkontinuität nach Harnröhrenverletzung) wurde im Verlauf im Rahmen des beckenosteosynthetischen Eingriffs urologischerseits empfohlen.

## Therapie und Verlauf

**Figure 2:**
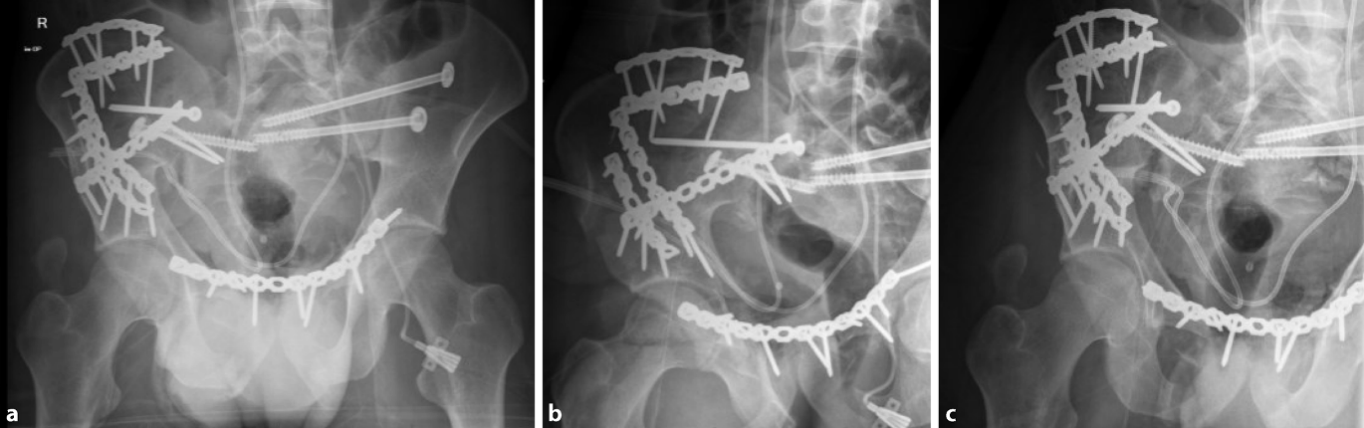

Die Versorgung der komplexen Beckenfraktur erfolgte zweizeitig. Im Rahmen des ersten Eingriffes am vierten posttraumatischen Tag, für den ein Stoppa-Zugang gewählt wurde, fand zudem die Rekonstruktion der Blasenwand und des Blasenhalses durch die Kollegen der Urologie statt (Abb. 2). Intraoperativ zeigte sich dabei ein nahezu vollständiger Abriss der Harnblase von der Prostata bis auf eine kleine dorsale Schleimhautbrücke. Es folgten die Harnblasenhalsrekonstruktion und Anastomosierung der Harnblase an die Prostata. Ein transurethraler Spülkatheter wurde zur Schienung der Urethra angelegt. Die zusätzliche Harnableitung über den suprapubischen Fistelkatheter wurde fortgeführt [4].

Im postoperativen Verlauf kam es wiederholt zur Urinextravasionen, welche mehrere urologische Revisionsoperationen nach sich zogen. Nach mehreren frustranen Versuchen, eine suffiziente Blasenhals-Harnröhren-Anastomose zu etablieren, musste schließlich ein Blasenhalsverschluss (inklusive Einlage von Harnleiterschienen bds. – Mono-J-Schienen) mit Einlage eines endständigen, suprapubischen Katheters durchgeführt werden. Bei weiterhin nachgewiesener Harnblasenleckage mussten trotz aller intensiven Bemühungen, die bereits teilweise nekrotische Harnblase zu erhalten, als Ultima Ratio die Zystektomie und Anlage eines Ileum-Conduits erfolgen. Dieses Vorgehen ist unüblich, jedoch bei einem so komplikationsreichen Verlauf alternativlos und eine individuelle Entscheidung. Ähnliche Fallberichte sind in der Literatur bisher nicht beschrieben.

**Figure 3:**
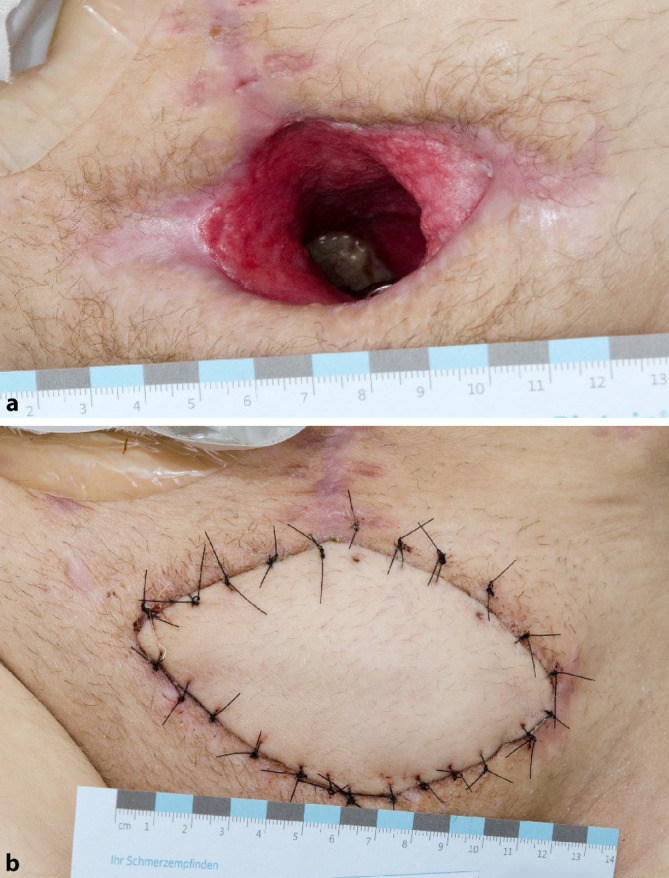

Der Patient entwickelte zudem eine Wundheilungsstörung auf Grundlage eines Wundinfektes. Mikrobiologisch wurde *Enterococcus faecium* nachgewiesen und resistenzgerecht antibiotisch behandelt. In Zusammenarbeit mit den Kollegen der plastischen Chirurgie wurde zunächst die temporäre Vakuumversiegelung durchgeführt und schließlich die Indikation zur Lappendeckung gestellt. Mittlerweile 2 Monate nach Unfall fand bei bereits fortgeschrittener knöcherner Durchbauung der vorderen Beckenringfraktur beidseits die Explantation der besiedelten Osteosynthese statt. In derselben Sitzung erfolgte der definitive Wundverschluss durch eine myokutane Lappenplastik vom linken Oberschenkel (Abb. 3).

Neurologisch bestätigte sich bei dem mittlerweile wachen und orientierten Patienten schließlich eine schwere rechtsbetonte inkomplette axonale Neuropathie des Plexus lumbosacralis. Klinisch imponierte dabei insbesondere eine Fußheberparese rechts, bei jedoch ebenfalls abgeschwächten Kraftgraden der übrigen Kennmuskeln. Mittlerweile anderthalb Jahre nach dem Unfall besteht klinisch ein nahezu unverändertes neurologisches Bild. Bei zeitgerechter Reinnervation der verletzten Nervenstrukturen ist mit einem Ende der axonalen Aussprossung jedoch erst insgesamt 3 Jahre nach dem Unfallgeschehen zu rechnen. Erst nach Ablauf dieser Zeit kann frühestens von einem Endzustand ausgegangen werden. Die noch bestehenden Ausfallerscheinungen sind aktuell mit der zeitgerechten Reinnervation in Verbindung zu bringen und lassen somit noch keine endgültige Beurteilung zu.

Zudem berichtete der Patient, mittlerweile über eine ungestörte Sexualfunktion (zufriedenstellende Erektionen und Ejakulationen) zu verfügen. Um die Lebensqualität des Patienten zu verbessern und die inkontinente in eine kontinente Harnableitung umzuwandeln, ist die Anlage einer Ileumersatzblase mit katheterisierbarem Nabelstoma geplant.

## Diskussion

In der Arbeit von Burkhardt et al. wurden die Daten des Beckenregisters mit denen des Traumaregisters abgeglichen [5]. Bei der Betrachtung schwer verletzter Patienten fand sich im untersuchten Zeitraum in 21 % der Fälle ein komplexes Beckentrauma. Die Inzidenz für Typ-C-Verletzungen des Beckens lag in diesem Patientenkollektiv doppelt so hoch wie bei den Patienten mit isolierter Beckenringfraktur, ohne die Kriterien eines komplexen Beckentraumas zu erfüllen. 97 % der Patienten wiesen einen ISS > 16 auf. Die Mortalität lag bei 16,7 % im Vergleich zu 5,9 % bei der Patientengruppe mit „regulärer“ Beckenringfraktur. Die Daten spiegeln letztlich die Brisanz dieser schweren Verletzung wider.

Die pelvinen Begleitverletzungen sind häufig äußerst komplex und stellen den behandelnden Unfallchirurgen vor große Herausforderungen. Nur im interdisziplinären Setting unter Nutzung aller Ressourcen ist ein optimales Behandlungsergebnis realisierbar [1, 2, 6, 9, 10, 14, 15, 18].

Das Retroperitoneum kann bis zu 6 l Blut aufnehmen. Neben dem Knochen als Hauptblutungsquelle sind der venöse präsakrale und prävesikale Venenplexus zu nennen. Es finden sich jedoch auch Blutungen aus dem Stromgebiet der A. iliaca interna sowie der A. iliolumbalis und A. glutea superior. Die Angaben zur Blutungshäufigkeit schwanken hier zwischen 0,6 und 27 % [8, 9, 13, 16]. Verletzungen der großen Gefäße machen eine chirurgische Intervention notwendig. Bei arteriellen Blutungen kommt der interventionellen Angiographie eine hohe Bedeutung zu [1, 5, 8, 10, 19].

Nervenverletzungen entziehen sich nicht selten der primären Diagnostik, da die Patienten oft intubiert und analgosediert in die Klinik gebracht werden. Schädigungen der nervalen Strukturen sind bei Beckenfrakturen in bis zu 50 % der Fälle anzutreffen [7, 9, 11]. Das Risiko steigt dabei mit der Schwere der Beckenverletzung bzw. mit dem Vorliegen einer Sakrumfraktur. Am häufigsten sind hier die Segmente L4 bis S2 betroffen, da sie aufgrund ihrer anatomischen Lage prädestiniert sind, bei entsprechenden Sakrumfrakturen mitgeschädigt zu werden.

Die Häufigkeit intraabdomineller Begleitverletzungen beim Beckentrauma schwankt in der Literatur zwischen 16 und 55 % [8, 12, 13].

Erwähnt sei in diesem Zusammenhang auch die Morel-Lavallée-Läsion, welche eine ausgedehnte Décollement-Verletzung der Weichteile beschreibt, wie sie häufig beim Überrolltrauma anzutreffen ist. Pohlemann et al. beschrieben hier eine Mortalitätsrate von 25 %. Primär ist die Décollement-Verletzung als mögliche Blutungsquelle als Todesursache mitverantwortlich. Sekundär kommt es jedoch häufig zur Infektion der betroffenen Weichteile und Hämatome bis hin zu Sepsis und Multiorganversagen [11].

Die Häufigkeit von Blasenverletzungen beim Beckentrauma wird mit 10–25 % angegeben. Das stumpfe Abdominaltrauma ist für 67–86 % aller Blasenrupturen verantwortlich. In 70–97 % sind diese assoziiert mit einer Beckenfraktur. Die Schweregradeinteilung der Blasenrupturen erfolgt über die AAST-Klassifikation [6, 17].

Bei den Blasenrupturen wird zwischen intraperitonealen und extraperitonealen Verletzungen unterschieden. Die extraperitoneale Blasenruptur ist immer mit der Verletzung des vorderen Beckenringes assoziiert [6, 7, 15, 17]. Die intraperitoneale Blasenverletzung ist Resultat eines sprunghaften intravesikalen Druckanstiegs bei hohem Füllungsvolumen als Folge einer erheblichen Krafteinwirkung auf das Becken und/oder untere Abdomen. Der Blasendom ist dabei der schwächste Punkt, sodass entsprechende Rupturen immer in diesem Bereich zu finden sind. Penetrierende Verletzungen sind auch hier die Ausnahme.

**Figure 4:**
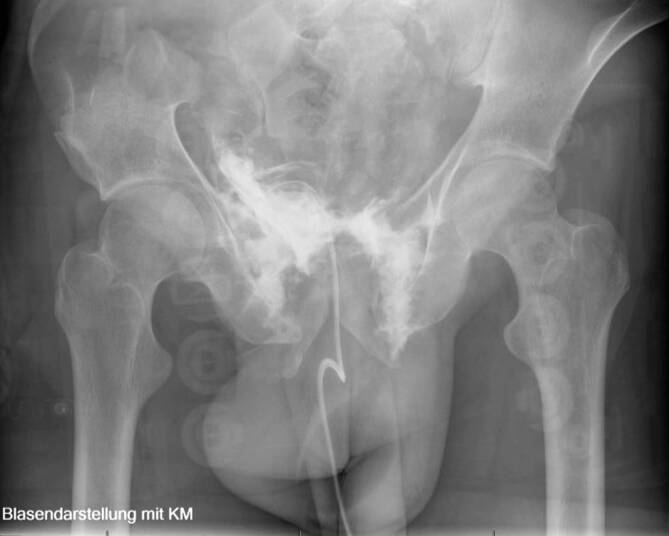

Als klinisches Zeichen sind hier die Makrohämaturie, Abdominalschmerzen, der Harnverhalt, suprapubische Hämatome, Schwellung des Skrotums oder Perineums zu nennen. Die Kombination aus Beckenfraktur und Makrohämaturie stellt die Indikation zur retrograden Zystographie. Bei unserem Vorgehen kommt die konventionelle Zystographie zur Anwendung, bei der mindestens 350 ml Kontrastmittel appliziert werden, gefolgt von einem Ablaufbild. Diese Untersuchungstechnik erreicht eine Sensitivität von 95 % mit einer Spezifität von 100 % (Abb. 4).

Die extraperitoneale Blasenruptur bei stumpfem Bauchtrauma kann meist konservativ behandelt werden. Sollte jedoch die Beckenringverletzung osteosynthetisch versorgt werden, wird auch die Naht der extraperitonealen Blasenruptur empfohlen, u. a., um das Infektionsrisiko zu senken.

Verletzungen mit Beteiligung des Blasenhalses, Knochenfragmenten in der Blasenwand oder intraperitoneale Rupturen müssen chirurgisch behandelt werden. Die intraperitoneale Urinextravasion kann sonst zu Peritonitis und Sepsis bis hin zum Tod führen [6, 17].

Urethraverletzungen sind in der Literatur in bis zu 15 % beschrieben. Besonders risikogefährdet sind dabei bilaterale Frakturen der Rr. ischiopubici. Auch hier erfolgt die Schweregradeinteilung nach der AAST-Klassifikation. Man unterscheidet zum einen partielle von vollständigen Urethrarupturen und zum anderen anteriore von dorsalen Wandverletzungen. Blut am Meatus ist das klinische Kardinalzeichen für eine Urethraverletzung. Allerdings schließt die fehlende Blutung auch keine Urethraverletzung aus. Die Unfähigkeit, willentlich zu urinieren (bei sonographisch oder palpatorisch gefüllter Harnblase), ist ein weiterer klassischer Hinweis für eine entsprechende Verletzung der Harnröhre. Häufig ist diese klinische Beobachtung mit einem vollständigen Urethraabriss vergesellschaftet [6, 14].

Die retrograde Urethrographie ist der Standard zur Diagnosesicherung. Jedes Kontrastmittelextravasat ist dabei pathognomisch für eine Urethraverletzung. Ein kompletter Urethraabriss ist dabei gekennzeichnet durch einen enormen Kontrastmittelaustritt ohne weiter Auffüllung der Blase. Ein typisches Zeichen für eine Partialruptur ist hingegen der Kontrastmittelaustritt, während sich die Blase noch weiter mit Kontrastmittel füllt. Beim weiblichen Geschlecht ist die retrograde Urethrographie aufgrund der kurzen Urethra nahezu unmöglich.

Läsionen der vorderen Harnröhre ohne assoziierte Penisruptur können häufig über eine transurethrale Schienung in Verbindung mit einer suprapubischen Harnableitung behandelt werden. Eine primäre offene Rekonstruktion ist mit einer erhöhten Rate an Impotenz und Inkontinenz verbunden und wird nicht empfohlen [6].

## Fazit für die Praxis

Das komplexe Beckentrauma ist ein seltenes und äußerst schweres Verletzungsbild und bedarf immer eines interdisziplinären Behandlungsansatzes. Unser Fallbeispiel ist in dieser Ausprägung und im geschilderten prolongierten, komplizierten Verlauf extrem selten, und das Management ist außerordentlich komplex.
